# CuCo and sulfur doped carbon nitride composite as an effective Fenton-like catalyst in a wide pH range

**DOI:** 10.3389/fchem.2022.982818

**Published:** 2022-08-24

**Authors:** Feifei Lin, Peng Liu, Rundong Lin, Chen Lu, Yuanyuan Shen, Yongqiang Wang, Xiwen Su, Hongjiang Li, Ying-Ying Gu

**Affiliations:** ^1^ Shandong Key Laboratory of Oil & Gas Storage and Transportation Safety, China University of Petroleum (East China), Qingdao, China; ^2^ College of Chemistry and Chemical Engineering, China University of Petroleum (East China), Qingdao, China; ^3^ College of Science, China University of Petroleum (East China), Qingdao, China; ^4^ Chengdu Hui Jin Water Development Co., Ltd., Chengdu, China; ^5^ School of Opto-electronic Engineering, Changchun University of Science and Technology, Changchun, China; ^6^ Qingdao Engineering Vocational College, Qingdao, China

**Keywords:** Fenton-like, bimetallic, hydrogen peroxide, organic pollutants, sulfur doped carbon nitride

## Abstract

The heterogeneous Fenton-like reaction, as an advanced oxidation process, is widely recognized attributed to its recyclability, wide pH response range, easy solid-liquid separation, and non-production of iron sludge. Recently, the bimetallic catalysts have attracted intense attention due to their high catalytic performance and excellent stability over a wide pH range. In this article, CuCo/SCN bimetallic catalyst was prepared by pyrolysis method with sulfur doped carbon nitride (SCN) as the carrier. Under the conditions of pH = 7, catalyst dosage of 0.8 g/L, and concentration of H_2_O_2_ of 15 mM, 20 mg/L of methyl orange (MO) can be completely removed within 1 h. With the synergistic action between bimetals and sulfur doped carbon nitride, the CuCO/SCN involved Fenton-like system exhibited excellent catalytic degradation efficiency and strong stability for MO in neutral and weak alkaline conditions. The EPR characterization proved that OH and O_2_^−^ were the main active components. Furthermore, CuCo/SCN involved Fenton-like system has good adaptability. Bimetallic CuCo/SCN catalyst has great application potential in the degradation of environmental pollutants.

## Introduction

With the rapid development of urbanization and industrialization, water pollution has become a major problem in the world, especially in developing and underdeveloped countries (Bethi et al., 2016; Wang et al., 2016; Liu et al., 2021). Various pollutants, such as industrial dyes, antibiotics, and endocrine disruptors, were discharged into surface water and groundwater annually, which can adversely affect human health and the ecosystem (Pavithra and Jaikumar, 2019; Lee et al., 2020; Qi et al., 2020). At present, researchers have adopted a variety of methods to treat contaminated water, such as flocculation, electrochemical, advanced oxidation (AOPs), and biological methods (Liu et al., 2014; Ike et al., 2019; Paździor et al., 2019). Among these methods, advanced oxidation shows great potential to convert most organic pollutants to smaller molecules or even to CO_2_ because of the highly efficient reactive oxygen species produced during the reaction (Dumrongrojthanath et al., 2013; Phuruangrat et al., 2015).

Fenton oxidation technology is one of the most cost-effective advanced oxidation technologies (Mark, 2008; Qian et al., 2017). In the classical homogeneous Fenton oxidation, Fe^2+^ activates H_2_O_2_ in an acidic environment to form strongly oxidizing OH, which destroys the pollutant structure without selectivity and forms intermediates (Yang et al., 2013). The resulting intermediates continue to react with OH and are completely decomposed into H_2_O and CO_2_. The homogeneous Fenton technology has the advantages of convenient operation, rapid reaction, and low cost. However, its operation pH is limited (generally 2.8–3.0), the utilization rate of hydrogen peroxide is low, and a large amount of sludge is formed (Mirzaei et al., 2017), restricting its practical application in wastewater treatment (Chen et al., 2011; Li et al., 2015; Ma et al., 2017). To solve these problems, researchers have developed and designed heterogeneous Fenton catalysis technology.

Iron-based Fenton catalysts are widely used in Fenton system because of their wide source, low cost, safety and harmlessness. However, the traditional iron-based Fenton catalysts also have some shortcomings, such as the need for strict pH regulation and the production of sludge, so the researchers turned their attention to other transition metals with Fenton catalytic activity and a wider range of pH adaptation (Lee et al., 2018; Zhu et al., 2019), such as Cu (Karthikeyan et al., 2016; Peng et al., 2019) and Co (Costa et al., 2006; Tu et al., 2012). In order to reduce metal dissolution, loading metals onto carriers is undoubtedly an efficient and convenient method. In recent years, carbon nitride has attracted people’s attention as a Fenton-like catalyst or carrier for heterogeneous Fenton-like catalysts due to its good stability, strong affinity for H_2_O_2_, good electron transfer performance, low cost and easy preparation (Liu et al., 2015; Liu et al., 2018; Pomilla et al., 2018). However, the shortcomings such as small specific surface area and long electron transfer path limit its application. It is therefore desirable to modify carbon nitride by doping metal or non–metal and hybridization with semiconductors to improve its catalytic activity and electron transfer performance in the field of catalysis (Yan et al., 2018; Han et al., 2019; Wang et al., 2019). In particular, the sulfur doped carbon nitride (SCN), which was prepared through a sulfur doping process, the introduction of low electronegative nonmetallic element sulfur can weaken the planar hydrogen bonding, replace marginalized NH/NH_2_ groups and improve electron transport efficiency. Furthermore, SO_2_ was generated during S pyrolysis, which changed the morphology of carbon nitride and increased the specific surface area (Ren et al., 2019). Consequently, the SCN is a potential carrier for Fenton catalyst.

In this study, a novel heterogeneous Fenton-like catalyst (CuCo/SCN) was fabricated with Cu and Co co-loaded onto a sulfur doped carbon nitride, which exhibits superior catalytic activity and good stability. The composite catalyst was characterized by Scanning electron microscopy (SEM), Transmission electron microscopy (TEM), X-ray diffraction (XRD), and X-ray photoelectron spectroscopy (XPS). And the degradation performance, stability, and adaptability of CuCo/SCN catalyst were evaluated. The main active components were also proved by the ESR characterization.

## Materials and methods

### Preparation of CuCo/SCN catalyst

**FIGURE 1:**
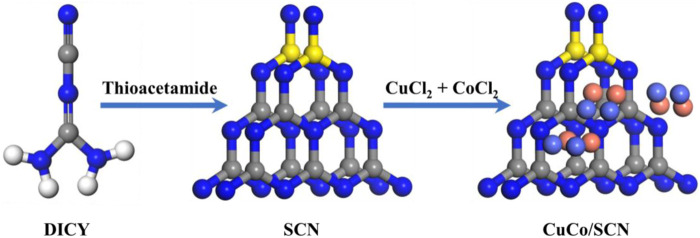
Preparation method of CuCo/SCN.

CuCo/SCN was prepared by pyrolysis as shown in Figure 1. Firstly, 6 g dicyandiamide (DICY) was transferred to 30 ml deionized water and stirred until the dicyandiamide was completely dissolved. 2 mmol CuCl_2_·2H_2_O, 2 mmol CoCl_2_·6H_2_O, and 50 mg of thioacetamide (TAA) were then added to the above solution. The mixed solution was heated in a 90°C water bath with continuous stirring, and the solid after drying was ground into powder. Finally, the obtained powder was pyrolyzed at 500°C in muffle furnace for 4 h, and the heating rate was 2.3°C/min. After cooling down to room temperature, the obtained powder was washed three times with deionized water to obtain the CuCo/SCN powder catalyst. Simultaneously, carbon nitride (CN), sulfur-doped carbon nitride (SCN), Cu/SCN and Co/SCN were prepared based on the same method, separately.

### Characterization of catalyst

SEM images and EDX spectroscopy elemental mapping analyses were obtained with a FEI model Quanta microscope at accelerating voltages of 15–30 kV. TEM images were obtained with a FEI Tecnai G2 microscope. XRD patterns were obtained with a Rigaku D/MAX 2400 instrument with a Cu source. XPS analyses were conducted with an Al Kα X-ray source (1486.6 eV) and a hemispherical concentric analyzer (CLAM2–VG Microtech). Fourier Transform Infrared Spectrometer (FTIR) (Brook Vertex 70) was used to identify the related functional groups in the catalyst.

### Fenton-like catalytic experiment

The effects of pH, H_2_O_2_ dosage, catalyst dosage, reaction temperature and pollutant concentration on the Fenton-like reaction were investigated and the optimum reaction conditions were determined. In order to study the stability and recycling of the catalyst in the process of use, the recycling experiment was carried out with methyl orange as a pollutant. Five cycle experiments were carried out, and the changes of degradation performance were measured. Rhodamine B, methylene blue, tetracycline and p-nitrophenol were selected as target pollutants to study the universality of the catalyst for the degradation of organic compounds, and to explore the degradation ability of the catalytic system for different pollutants.

## Results and discussion

### Characterization of catalyst

**FIGURE 2:**
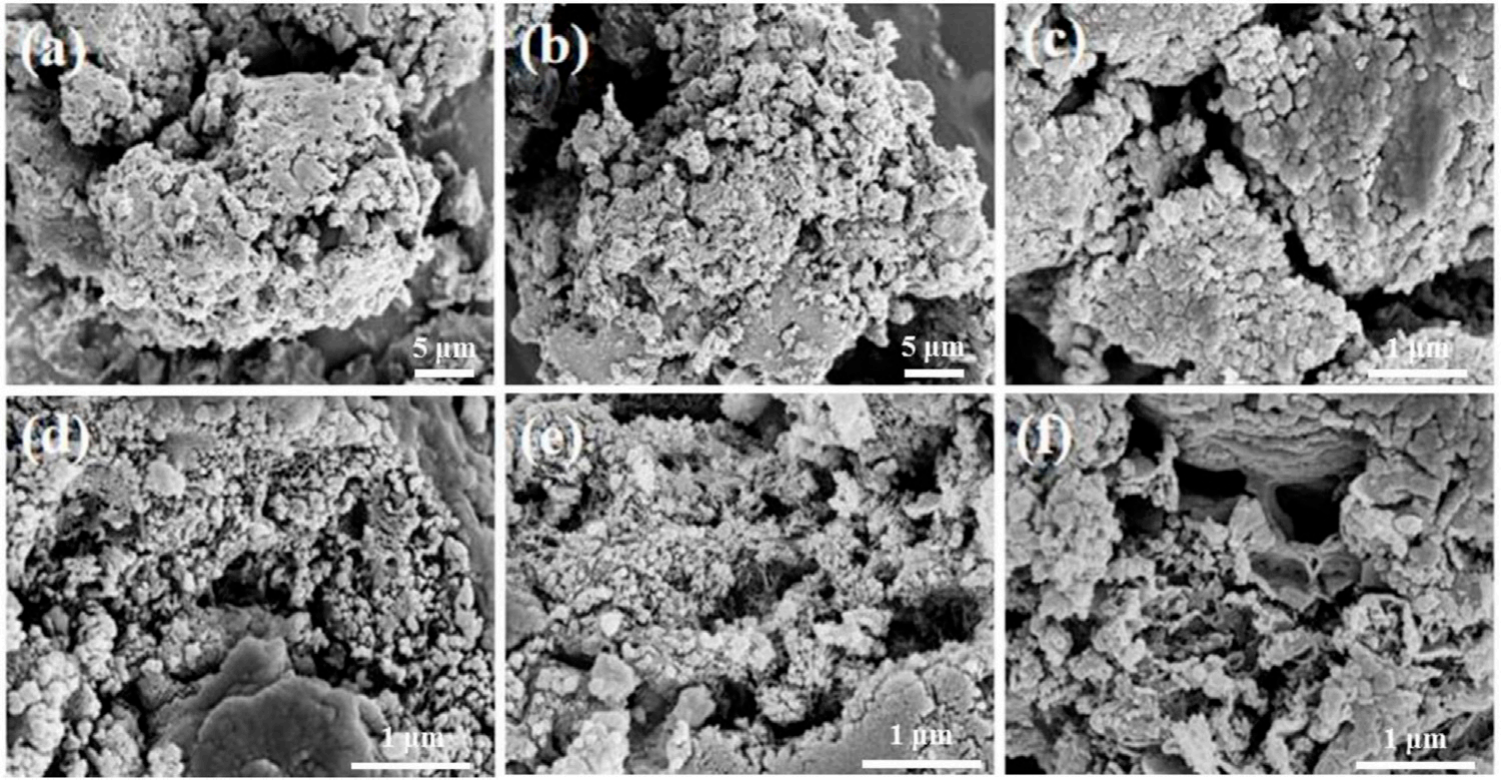
SEM images of **(A)** CN, **(B)** SCN, **(C)** Cu/SCN, **(D)** Co/SCN, **(E)** CuCo/CN, and **(F)** CuCo/SCN.

The micro-morphology of the prepared samples was studied by SEM images as shown in Figures 2A,B, pure CN is an irregularly arranged bulk structure with agglomerated structure, compact morphology and rough surface. After doping with S, the surface becomes fluffy, but the bulk structure did not change significantly. There are highly dispersed granular substances with different sizes on the surface as shown in Figures 2C–F. When the SCN is loaded with metal, fine metal particles are deposited on the SCN surface and are highly dispersed. When the bimetals are loaded together, the catalyst surface shows a porous structure. This is because the addition of Cu and Co in pyrolysis accelerates the deamination process, and the gas produced by deamination makes the catalyst form porous structure. The BET characterization (Supplementary Table S1) also demonstrated that sulfur doping and metal loading contributed to the increase of the specific surface area.

**FIGURE 3:**
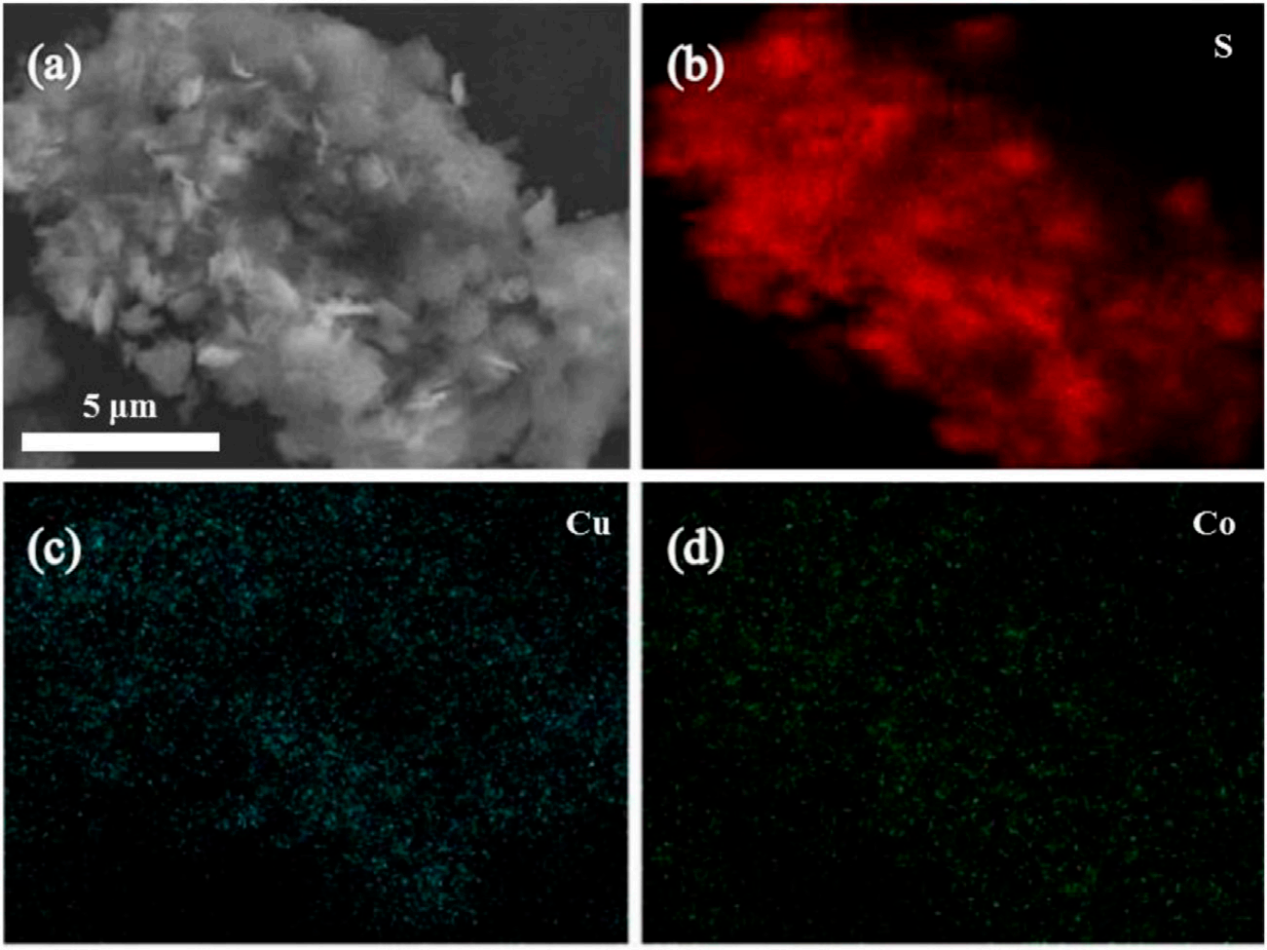
**(A)** SEM image of CuCo/SCN and element mapping of **(B)** S, **(C)** Cu, **(D)** Co.

The elemental mapping analysis of the catalyst as shown in Figure 3 showed that S, Cu, and Co elements are uniformly dispersed on the surface of the catalyst material, which indicates that Cu and Co have been successfully combined with sulfur-containing carbon nitride, and this result is consistent with the SEM image.

**FIGURE 4:**
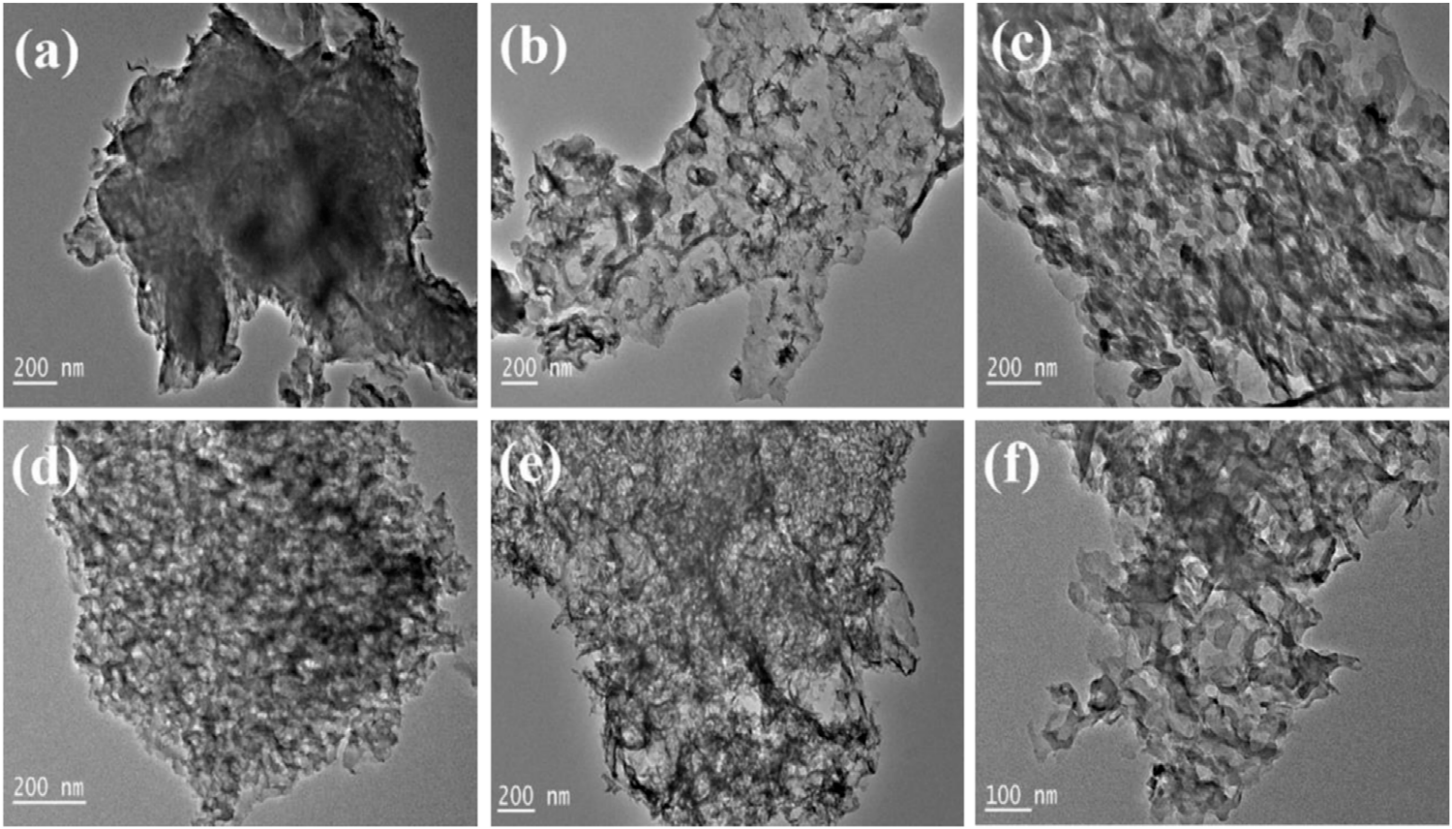
TEM images of **(A)** CN, **(B)** SCN, **(C)** Cu/SCN, **(D)** Co/SCN, **(E)** CuCo/CN, and **(F)** CuCo/SCN.

It is shown in Figure 4A that the pristine CN is formed by stacking of lamellar structures. After doping with S, the color of the SCN sheet is much more transparent as shown in Figure 4B, which may be attributed to the influence of sulfur element on the hydrogen bonds between the CN layers, which weakens the hydrogen bonds, indicating that the layered structure becomes thinner due to the introduction of S. Relevant studies have pointed out that the flake-like carbon nitride is beneficial to shorten the electron transport distance and improve the catalytic activity of the reaction. Figures 4C–F shows that Cu and Co are successfully loaded and uniformly dispersed on the SCN substrate. The bimetallic loading creates more pores on the SCN surface, increasing the active sites and improving the catalytic performance.

**FIGURE 5:**
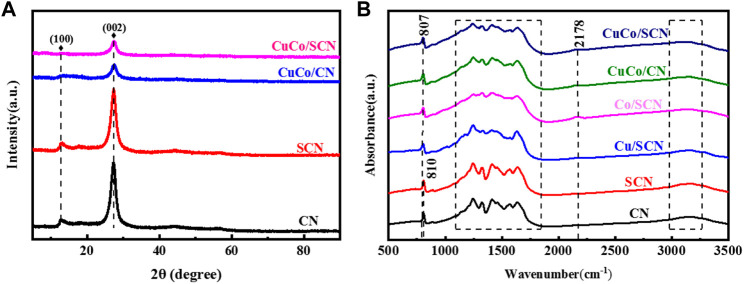
**(A)** XRD patterns and **(B)** FTIR patterns of the samples.

The crystal form and crystallinity of the catalyst were analyzed by XRD pattern. In Figure 5A, pure CN exhibits characteristic diffraction peaks at 13.1° and 27.4°. Among them, the diffraction peak at 13.1° corresponds to the (100) crystal plane of the planar ordered tris-triazine ring unit, and the diffraction peak at 27.4° corresponds to the (002) crystal plane, which is caused by the accumulation of the conjugate system, indicating that the as-prepared CN possesses a layered structure similar to that of graphite. There are similar diffraction peaks before and after sulfur doping, which indicates that the crystal structure of CN would not change after doping of sulfur. The characteristic diffraction peaks of Cu and Co were also not found in the CuCo/SCN samples, indicating that Cu and Co would not form corresponding crystal structures in SCN, or the crystal size of metal and metal oxide formed was less than 4 nm and highly dispersed, resulting in weak signal. However, the diffraction peak intensities of the CuCo/SCN samples at 13.1° and 27.4° were significantly weakened after loading Cu and Co, which may be caused by the incomplete polymerization of the metal precursor during the pyrolysis process, or it may be due to the loading of the metal. The lattice of the original carbon nitride undergoes subtle changes, which affected the growth of the grains.

FTIR spectrum analyses (Figure 5B) identified the relevant functional groups in the catalyst. The peak at 810 cm^−1^ in the pure CN sample is caused by the bending vibration of the triazine ring. When the metal is loaded, the characteristic peak shifts slightly to 807 cm^−1^. The region from 1,100 to 1,650 cm^−1^ is the characteristic absorption band of the stretching vibration of aromatic CN heterocycles. The diffraction peak at 1640 cm^−1^ is attributed to the stretching vibration of water and hydroxyl adsorbed on the carbon nitride surface. The peak at 3,000–3,500 cm^−1^ corresponds to the amino terminal functional group and belongs to the stretching vibration of N-H bond, indicating that the polycondensation of dicyandiamide after pyrolysis is incomplete, and there is still N-H bond at the edge of layered CN. However, the stretching vibration obviously related to sulfur has not been found in the spectrum of SCN samples, which may be caused by the relatively low sulfur content. The peaks at 1,100–1,650 cm^−1^ are weakened after the introduction of metal, which may be due to the complexation of Cu and Co with CN, which inhibits the polymerization process of some precursors and affects the stretching vibration of CN materials. In all the prepared samples, the characteristic absorption peaks of the breathing vibration of the triazine ring and the stretching vibration of the aromatic CN heterocycle still exist, indicating that all the samples retain the basic skeleton of CN.

**FIGURE 6:**
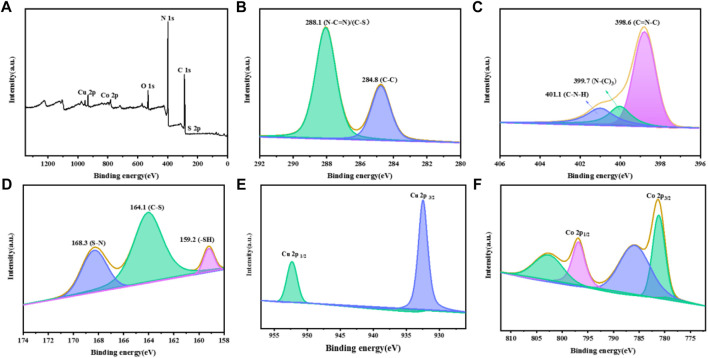
XPS patterns of the CuCo/SCN: **(A)** Survey, **(B)** C 1s, **(C)** N 1s, **(D)** S 2p, **(E)** Cu 2p, and **(F)** Co 2p.

The XPS spectra of CuCo/SCN samples are shown in Figure 6. Figure 6A is the survey spectrum of the sample, where the characteristic peaks of C, N, O, S, Cu, and Co appear. The characteristic peaks appear at 288.1 and 284.8 eV in the C 1s spectrum of the sample, corresponding to sp2 hybrid bond (N-C=N) and graphite like carbon (C-C), respectively. The characteristic peaks of C-S and C-N overlap. The N 1s spectrum in Figure 6C can be fitted into three characteristic peaks. 398.5 eV belongs to C-N=C, 399.4 eV belongs to N-(C)_3_, and 400.8 eV belongs to C-N-H, which may be caused by incomplete polymerization and structural defects. As shown in Figure 6D, the characteristic peaks at 159.2, 164.1, and 168.3 eV belong to -SH, C-S, and S-N, respectively in the S 2p spectrum. In the spectrum of Cu 2p (Figure 6E), the peaks at 932.4 and 952.3 eV are attributed to Cu^+^ 2p_3/2_ and 2p_1/2_, respectively. The spectrum of Co 2p is shown in Figure 6F. The two characteristic peaks at 781.1 and 795.9 eV correspond to Co^2+^ 2p_3/2_ and Co^2+^ 2p_1/2_, respectively, while the two peaks at 785.9 and 802.9 eV correspond to Co^3+^ 2p_3/2_ and Co^3+^ 2p_1/2_.

### Single factor degradation experiment

**FIGURE 7:**
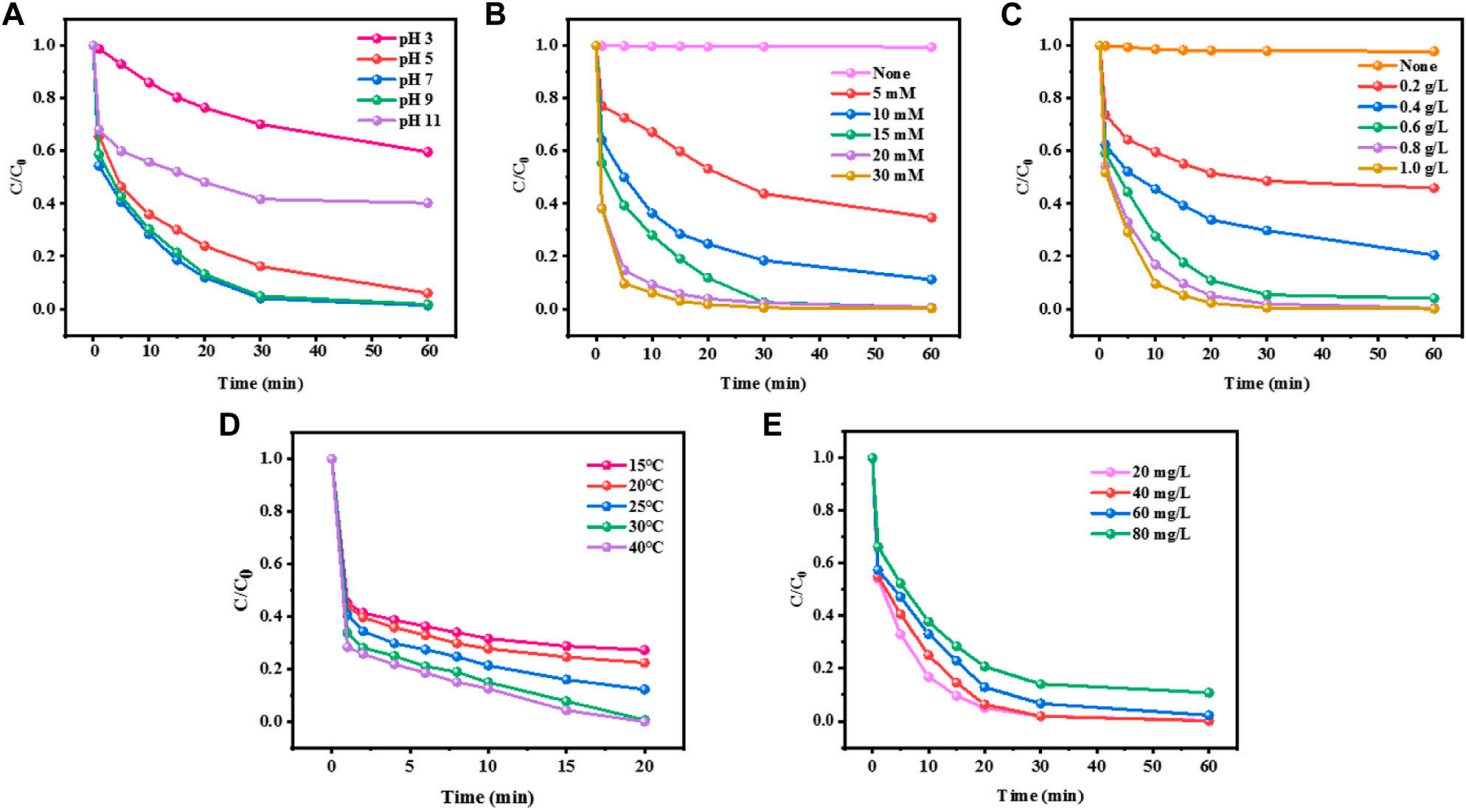
Effect of **(A)** initial pH, **(B)** H_2_O_2_ dosage, **(C)** catalyst dosage, **(D)** reaction temperature, and **(E)** initial MO concentration on the removal of MO.

For the traditional iron-based homogeneous Fenton reaction, pH is a crucial factor, which mainly reflects that pH determines the hydrolysis form of Fe (Ⅱ), thus affecting the reaction between H_2_O_2_ and Fe (Ⅱ). The currently recognized optimum pH value of the Fenton reaction is about 3.0, which greatly limits the practical application of the Fenton reaction. The extremely acidic operating conditions not only increase the operating cost, but also bring corrosion to the equipment. The effect of solution pH on the catalytic degradation of MO in the CuCo/SCN involved Fenton-like reaction system was investigated in Figure 7A. The experimental conditions were as follows: reaction temperature: 30°C, initial concentration of Mo: 20 mg/L, H_2_O_2_ concentration: 15 mM, and CuCo/SCN dosage: 0.8 g/L. When pH was 7, CuCo/SCN catalyst showed good catalytic activity, and all pollutants can be removed within 60 min. Under the conditions of weak acidic (pH = 5) and weak alkaline (pH = 9), the removal rates can reach 92% and 100%, respectively. Under strong acidic conditions (pH = 3), its catalytic ability was inhibited, and only 35% of pollutants were removed after 60 min. The reason may be that the catalyst is unstable under acidic conditions, which leads to the leaching of metal ions. In addition, excessive H^+^ not only reacts with H_2_O_2_ to generate hydrated hydroxyl ion (H_3_O_2_^+^) (Eq. 1), which reduces the utilization rate of H_2_O_2_, but also consumes OH (Eq. 2). When pH is 11, only 59% can be removed by oxidation in 60 min. The reason is that H_2_O_2_ is very easy to decompose ineffectively under alkaline conditions (H_2_O_2_ reacts with strong alkali to form HOO-, which has poor stability), which reduces the output of OH and directly affects the degradation of pollutants. The CuCo/SCN catalyst exhibited the best catalytic performance at pH 7. Compared with traditional catalysts, CuCo/SCN has the best catalytic activity under neutral conditions, and even shows good treatment effect under weak alkaline conditions. Moreover, the isoelectric point by Zeta Potential at different pH values was determined for the CuCo/SCN (Supplementary Table S2). The composite CuCo/SCN presented a negative charge surface for the pH values tested (7, 9, and 11), being less intense at neutral and weakly alkaline medium, which makes it easier for the molecules of the contaminant to be adsorbed on the composite surface, resulting in a greater decomposition of said molecules. This has important research significance for improving the stability of the catalyst, broadening the optimal pH range, saving treatment cost and popularizing the practical application of Fenton reaction.

$$H_{2}O_{2}+H^{+}\to H_{3}O_{2}^{+}$$ (1)

$$\cdot OH+H^{+}+e^{-}\to H_{2}O$$ (2)

As the oxidant of the reaction system, H_2_O_2_ is the source of OH, and its dosage will directly affect the performance and cost of the Fenton system. Therefore, it is necessary to explore the optimal concentration of H_2_O_2_. The effect of H_2_O_2_ concentration on the catalytic degradation of Mo in CuCo/SCN involved Fenton-like reaction system was investigated under other experimental conditions, and the results were shown in Figure 7B. MO in the system could not degrade obviously without H_2_O_2_, which indicated that CuCo/SCN had no degradation ability and very small adsorption ability. With the increase of H_2_O_2_ concentration from 5 to 15 mM, the degradation efficiency of Mo increased from 65.4% to 99%. However, when the H_2_O_2_ dosage was further increased to 20 and 30 mM, the catalytic removal effect of MO could not change significantly. The low concentrations of H_2_O_2_ produce insufficient OH, and the pollutants cannot be completely degraded. As the amount of H_2_O_2_ increased within a certain range, the rapid reaction between CuCo/SCN and H_2_O_2_ produced more OH, which improved the reverse degradation effect of MO.

The dosage of catalyst is also an important parameter to evaluate the catalytic activity of catalyst. Figure 7C showed that the effect of catalyst dosage on the catalytic degradation of MO in CuCo/SCN involved Fenton-like reaction system was investigated. MO was hardly removed without CuCo/SCN added, which indicated that the oxidation ability of H_2_O_2_ alone was not enough to degrade the pollutant MO. When the dosage of CuCo/SCN was 0.2 g/L, the removal rate of MO was only 50% after 60 min. When the dosage increased to 0.6 g/L, the removal rate of MO increased to 96%. When the dosage continued to increase to 0.8 g/L, MO was completely removed. However, when the dosage was increased to 1 g/L, the removal rate changed little. The results showed that increasing the amount of catalyst is beneficial to the degradation of MO in a certain range. With increasing the amount of catalyst, the catalyst in Fenton system can provide more contact reaction surfaces to increase the probability of solid-liquid contact between H_2_O_2_ and CuCo/SCN, thus more OH produced to significantly improves the removal efficiency of MO. Excessive addition of catalyst will not only increase the treatment cost, but also increase the risk of metal dissolution and cause secondary pollution.

The reaction temperature is also the key factor to evaluate the degradation performance of catalyst. The degradation effect of CuCo/SCN catalyst at different reaction temperatures was shown in Figure 7D. When the reaction temperature was 15°C, the removal rate of the MO was only 70%, and when the temperature was raised to 25°C, 86% of MO was degraded, and when the temperature continued to 30°C, MO was completely decolorized. The higher reaction temperature accelerates the Fenton reaction process of the CuCo/SCN involved Fenton-like system to generate OH, thereby rapidly oxidizing and degrading pollutants. Meanwhile, the higher reaction temperature can also promote the diffusion of OH and improve the catalytic performance of the CuCo/SCN catalyst. However, high temperature may decompose H_2_O_2_, reduce the utilization rate of H_2_O_2_, affect the adsorption of pollutants on CuCo/SCN surface and hinder the Fenton reaction process.

As shown in Figure 7E, different concentrations of MO from 20 to 80 mg/L were degraded by the CuCo/SCN catalyst. Even when the concentration of MO reached 80 mg/L, about 90% of the pollutants could be degraded within 60 min, indicating excellent performance of the catalyst. Based on the above results, the optimal experimental conditions for the degradation of MO by CuCo/SCN catalyst Fenton are as follows: the reaction temperature is 30°C, the pH value is 7, the initial MO concentration is 20 mg/L, the H_2_O_2_ concentration is 15 mM, and the catalyst dosage is 0.8 g/L. Meanwhile, the degradation effects of CN, SCN, Cu/SCN, Co/SCN and CuCo/SCN were compared under the optimal conditions (Supplementary Figure S1), and CuCo/SCN showed the best degradation performance.

### Study on the stability of catalyst

**FIGURE 8:**
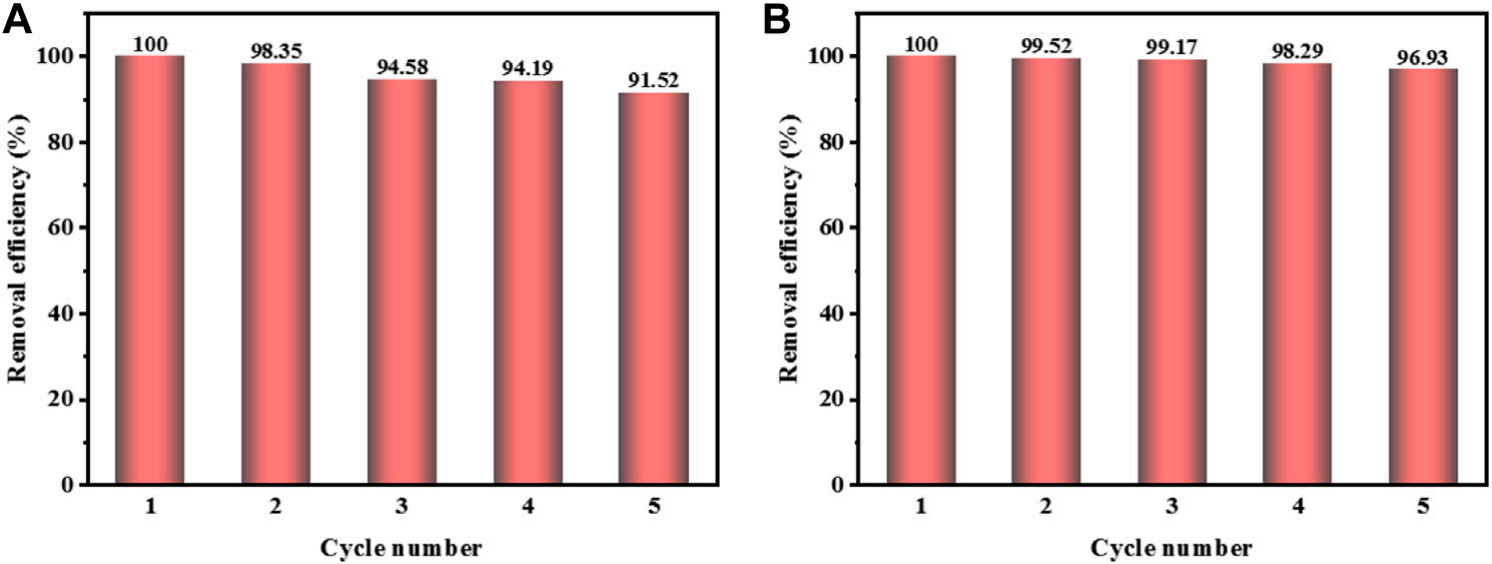
5 cycles of reuse test: **(A)** pH = 7, **(B)** pH = 9.

Stability is an important index to identify the catalytic performance of Fenton catalyst. In this study, CuCo/SCN catalyst was tested for five cycles at pH = 7 and 9 (Figure 8), respectively. The CuCo/SCN catalyst exhibited high stability under neutral and weak alkaline conditions. After 5 cycle experiments, the degradation efficiency of MO decreased from 100% to 91.52% and 96.93%, respectively.

**FIGURE 9:**
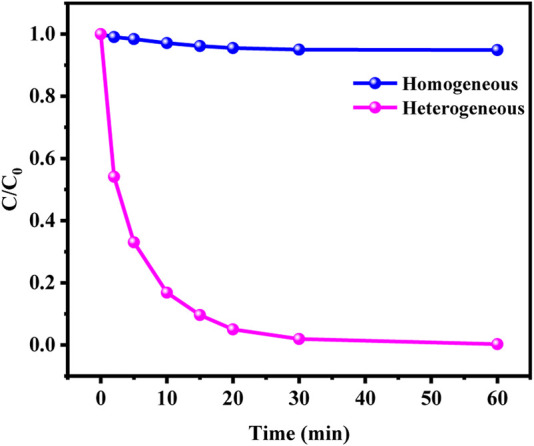
Homogeneous/Heterogeneous Fenton degradation of MO.

The metal leaching after reaction was analyzed by ICP. The results showed that the leaching efficiencies of Cu and Co were 0.08% and 0.3% respectively. Such a small leaching efficiency further proved that Cu and Co are effectively fixed on SCN under the action of chemical bond. The removal effects of homogeneous and heterogeneous Fenton reaction were compared to explore the degradation of leached copper and cobalt ions in homogeneous Fenton reaction. As shown in Figure 9, the homogeneous copper and cobalt ions released into the solution have little effect on the degradation of MO in the CuCo/SCN involved Fenton-like system, indicating that the degradation of MO is attributed to the heterogeneous Fenton reaction of the CuCo/SCN involved Fenton-like catalyst.

**FIGURE 10:**
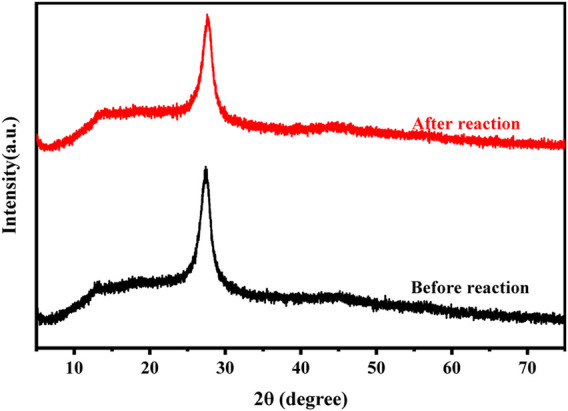
XRD patterns of CuCo/SCN before and after reaction.

The XRD spectrums of CuCo/SCN catalyst before and after Fenton reaction as shown in Figure 10. The peak intensity of CuCo/SCN catalyst at 27.4° was only slightly weakened after Fenton reaction, which was attributed to the slight decrease in the crystallinity of carbon nitride after catalytic reaction. However, there are consistent diffraction peaks before and after the reaction, and no impurity peaks are generated after the reaction. The XRD results show that the chemical structure and crystal form of CuCo/SCN have almost no change after being reused for 5 times, which further verifies the better stability of CuCo/SCN.

### Analysis of catalytic mechanism

**FIGURE 11:**
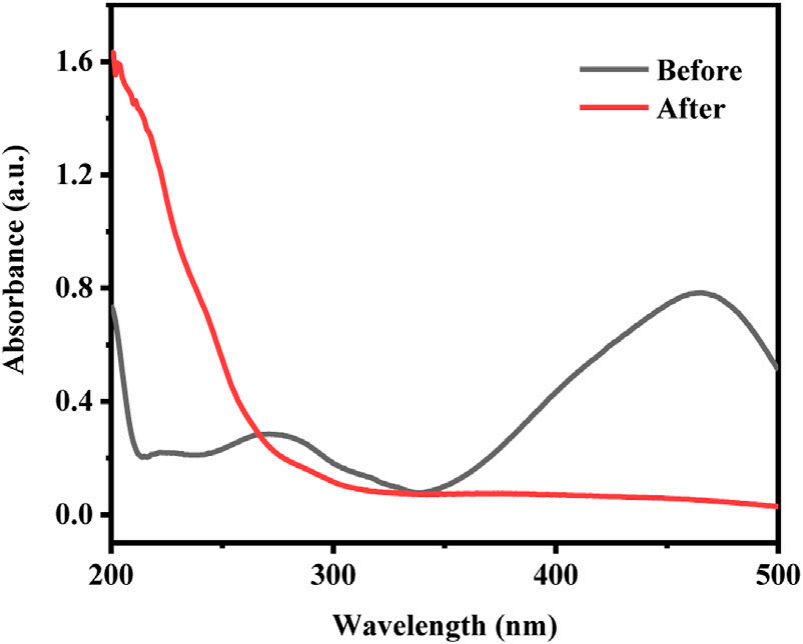
| The UV-Vis absorption spectra of the reaction solution before and after Fenton reaction.

In order to understand the mineralization degree of MO, the total organic carbon (TOC) of MO as the target pollutant was tested under the same reaction conditions. The results showed that 41.1% of TOC can be removed by CuCo/SCN involved Fenton-like system after 60 min, indicating that CuCo/SCN involved Fenton-like system can effectively mineralize MO. However, the degree of MO mineralization is incomplete, which may be attributed to the colorless intermediate small molecule products generated by the Fenton reaction. The UV-Vis absorption spectra of the reaction solution before and after Fenton reaction were tested to verify the above view. As shown in Figure 11, the maximum absorption wavelength of methyl orange was at 463 nm, which was attributed to the characteristic peak of the chromophore of -N=N- double bond. There was another characteristic peak at 271 nm, corresponding to the characteristic peak of benzene ring and its homologues. In the UV-vis absorption spectra of the reaction solution after Fenton reaction, the absorption peak of methyl orange at the wavelength 463 nm had completely disappeared, which indicated that the chromogenic group of methyl orange had been destroyed. Meanwhile, the characteristic peak in the ultraviolet region was also transferred from the initial 271–212 nm, and there was a new characteristic peak at 204 nm. The above results showed that the methyl orange wastewater treated by Fenton oxidation can be decolorized completely, which was consistent with the decolorization rate in the specific experiment. However, the solution still had characteristic absorption peaks in the ultraviolet region after the reaction, indicating that there were still benzene rings and their homologues in the waste liquid. Fenton oxidation only partially oxidizes methyl orange macromolecules into colorless intermediate small molecular products, but could not completely mineralize pollutants into CO_2_ and H_2_O.

**FIGURE 12:**
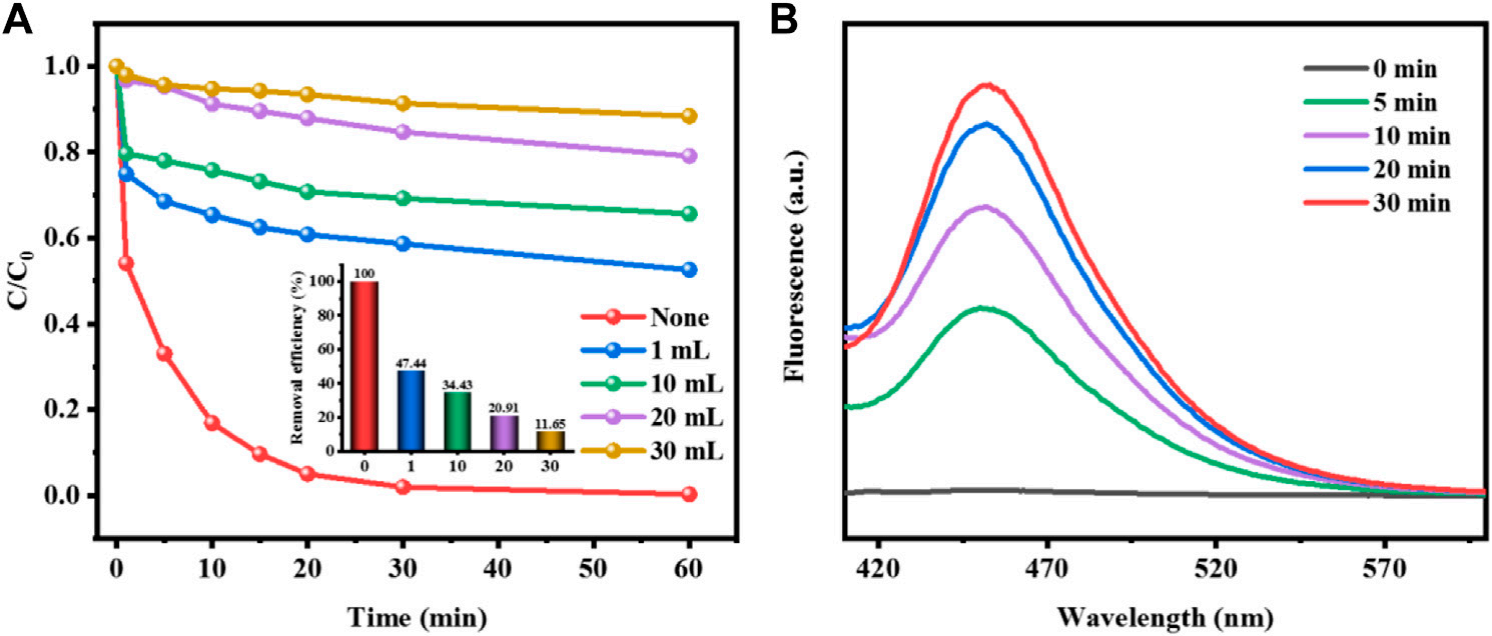
**(A)** Effect of the amount of isopropanol on MO removal efficiency, **(B)** Fluorescence intensity of 7-hydroxycoumarin during the reaction.

During the Fenton-like reaction for removing pollutants, the catalyst activates H_2_O_2_ to produce hydroxyl radicals which attack organic pollutants. The free radical quenching experiment was used to explore and analyze the active species in the degradation of methyl orange by CuCo/SCN catalyst. According to the literature, isopropanol (IPA) could effectively capture OH and slow down or terminate the degradation reaction. Under the same experimental conditions, different amounts of free radical remover were added to the reaction system to compare the pollutant removal efficiency and select the best dosage. As shown in Figure 12A, the addition of IPA greatly inhibited the degradation efficiency of methyl orange. Without IPA, the decolorization rate of methyl orange could reach 100%. With the increase of methyl orange dosage, the degradation efficiency of IPA decreased sharply. The quenching experiment confirmed that OH was the main active substance of CuCo/SCN involved Fenton-like reaction system. H_2_O_2_ is catalytically reduced to OH by CuCo/SCN, which can efficiently degrade and mineralize pollutants. Coumarin can react with OH to produce fluorescent substance 7-hydroxycoumarin. Therefore, to verify the yield of OH in the reaction system, coumarin is added to the reaction system, and the fluorescence spectrum of 7-hydroxycoumarin in the reaction solution is detected by fluorescence spectrophotometer. Figure 12B shows the fluorescence intensity spectrum of 7-hydroxycoumarin during the reaction. With the progress of the reaction, the fluorescence spectrum intensity increased, indicating that the amount of OH produced by H_2_O_2_ catalyzed by CuCo/SCN also increased.

**FIGURE 13:**
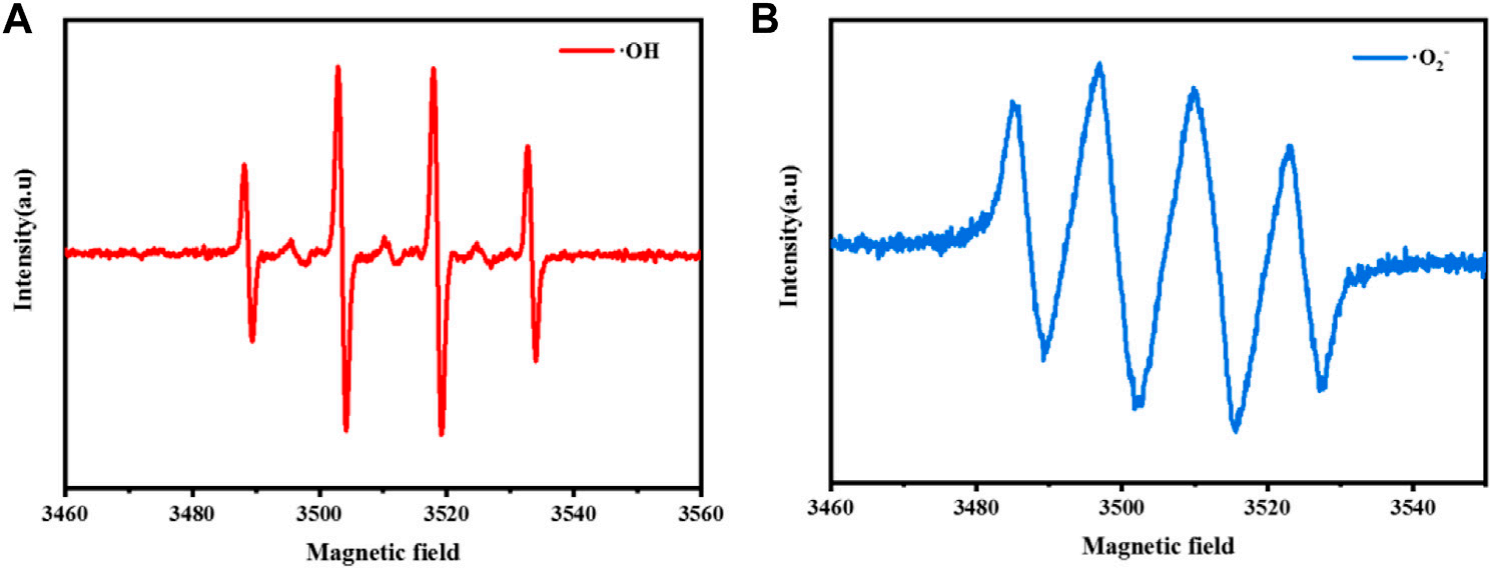
EPR characterization of CuCo/SCN involved Fenton-like system: **(A)** DMPO-·OH, **(B)** DMPO-·O_2_^−^.

In order to verify the above conclusions, this paper determines the free radical species produced in the reaction system by Dimethyl pyridine N-oxide (DMPO)—electron spin resonance (ESR) technology. As shown in Figure 13, DMPO captured ESR signal of OH and O_2_^−^ ESR signal in CuCo/SCN sample suspension medium. The H_2_O_2_ is selectively reduced to OH and attack contaminants, and O_2_^−^ can also assist degradation. The above results showed that the prepared CuCo/SCN can quickly and effectively reduce H_2_O_2_ to produce hydroxyl radicals with stronger oxidation activity, which promotes the degradation of pollutants.

### Adaptability of the catalyst

**FIGURE 14:**
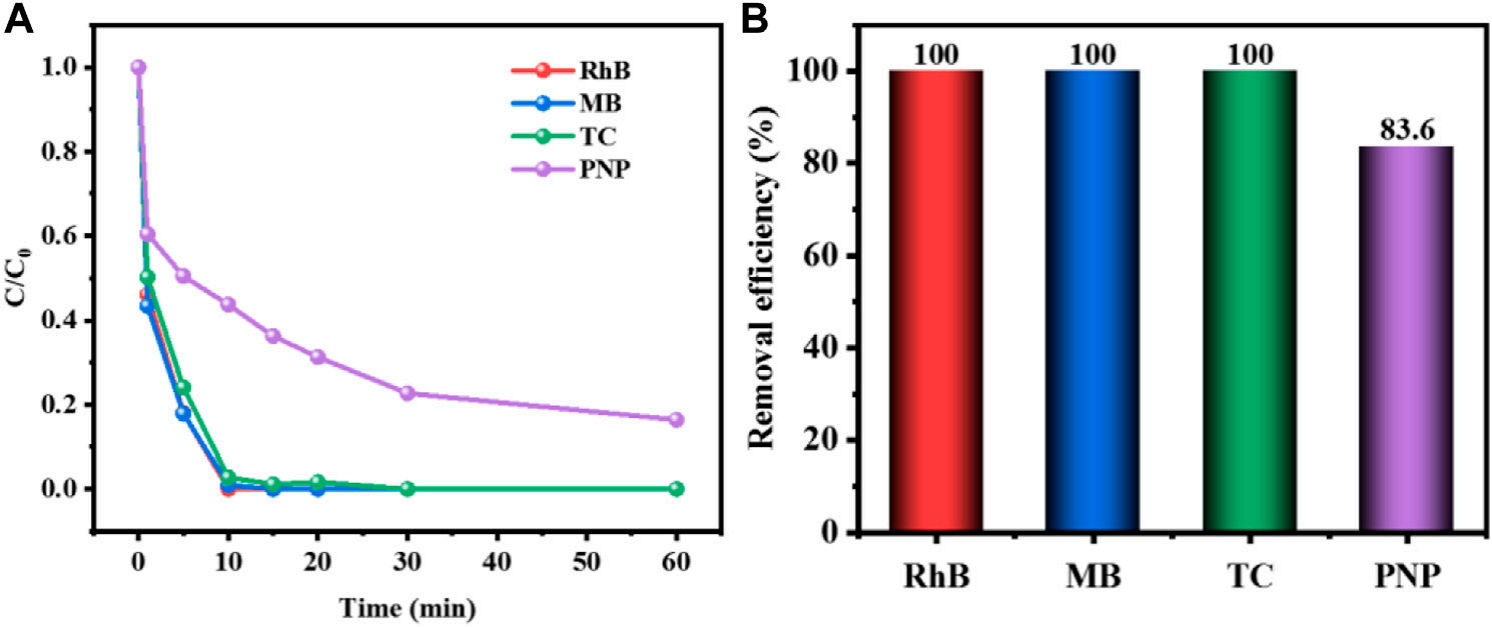
**(A)** Degradation curve and **(B)** removal efficiency of CuCo/SCN involved Fenton-like system for other organic pollutants.

To explore the adaptability of CuCo/SCN catalyst, CuCo/SCN involved Fenton-like system was used to degrade other organic pollutants, including RhB, MB, TC, and PNP. The results in Figure 14 showed that the removal efficiency of RhB, MB, and TC could reach 100% in 1 h, and the degradation efficiency of PNP could also reach 83.6% in 1 h. The experimental results showed that CuCo/SCN involved Fenton-like system has a good removal effect on all kinds of target pollutants selected in the experiment, and it also proves that CuCo/SCN catalyst has good Fenton Catalytic Performance.

## Conclusion

In this study, bimetallic CuCo/SCN catalysts were prepared by pyrolysis method using dicyandiamide as carbon nitride precursor, thioacetamide as sulfur precursor, and copper chloride and cobalt chloride as raw materials. The best bimetallic CuCo/SCN catalyst was selected by investigating the metal ratio and calcination temperature. Bimetallic CuCo/SCN catalyst showed excellent Fenton degradation performance and catalytic stability under neutral and weak alkaline conditions. Meanwhile, CuCo/SCN involved Fenton-like system had good universality and could degrade a variety of organic pollutants, such as MO, RhB, MB, TC, and PNP. The preparation of bimetallic CuCo/SCN catalyst broadens the application range of Fenton reaction and provides a new idea for the current environmental pollution control.

## Data Availability

The original contributions presented in the study are included in the article/Supplementary Material, further inquiries can be directed to the corresponding authors.
