# Supplementary figures and images for: CuCo and sulfur doped carbon nitride composite as an effective Fenton-like catalyst in a wide pH range
Source: Front Chem. 2022 Aug 24;10:982818. doi: 10.3389/fchem.2022.982818 (PMC9449145; doi:10.3389/fchem.2022.982818)

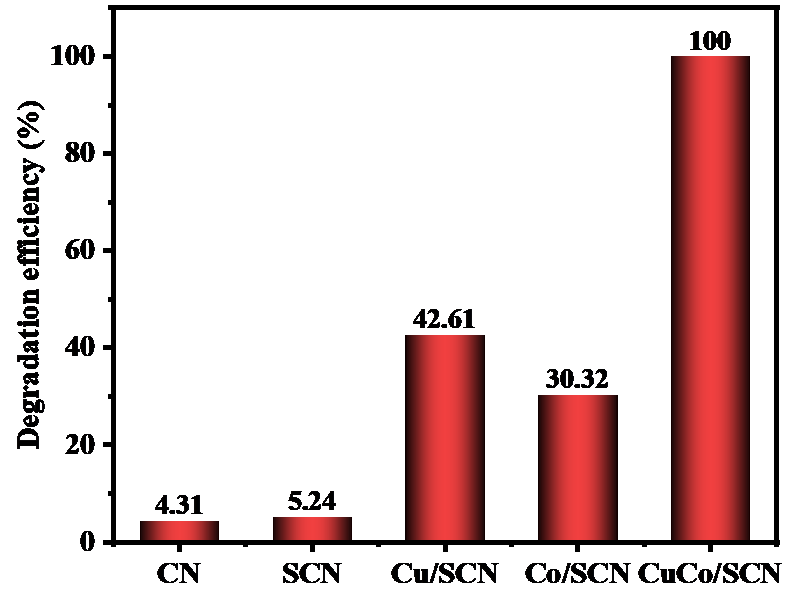

Supplement: Supplementary file 1 [file Image1.PNG]
