# Supplementary material for: CuCo and sulfur doped carbon nitride composite as an effective Fenton-like catalyst in a wide pH range
Source: Front Chem. 2022 Aug 24;10:982818. doi: 10.3389/fchem.2022.982818 (PMC9449145; doi:10.3389/fchem.2022.982818)
Supplement: Supplementary file 2 [file DataSheet1.docx]

**CuCO and Sulfur doped carbon nitride composite as an effective Fenton-like catalyst in a wide pH range**

**Lin Feifei^1,2,†^, Liu Peng^3,†^, Lin Rundong^4^, Lu Chen^2^, Shen Yuanyuan^2^, Wang Yongqiang^2^,** **Su Xiwen^5,*^, Li Hongjiang^6^, Gu Ying-Ying^1,2,*^**

^1^ *Shandong Key Laboratory of Oil & Gas Storage and Transportation Safety, China University of Petroleum (East China), Qingdao 266580, China.*

^2^ *College of Chemical Engineering, China University of Petroleum (East China), Qingdao 266580, China.*

^3^ *College of Science, China University of Petroleum (East China), Qingdao 266580, China.*

^4^ *Chengdu Hui Jin Water Development Co. Ltd., Chengdu 611730, China.*

^5^ *Changchun University of Science and Technology, Changchun, 130022, China.*

^6^ *Qingdao Engineering Vocational College, Qingdao 266580, China.*

ǂ These authors have contributed equally to this work and share first authorship

*** Correspondence:**Su Xiwen
suxinwenemma@qq.com;
Gu Ying-Ying
yingyinggu@upc.edu.cn.

**Table S1** The BET characterization of the different Fenton catalysts.

| Samples | Surface Area  (m^2^·g^-1^) | Pore Diameter  (nm) | Pore Volume  (cm^3^·g^-1^) |
| --- | --- | --- | --- |
| CN | 7.98 | 9.82 | 0.03 |
| SCN | 8.01 | 10.83 | 0.03 |
| CuCo/CN | 12.17 | 14.65 | 0.04 |
| CuCo/SCN | 13.95 | 15.63 | 0.05 |

**Table S2** Variation of the Zeta Potential of the CuCo/SCN with different pH values.

| pH | Zeta potential (mV) |
| --- | --- |
| 3 | 42.3 |
| 5 | 12.4 |
| 7 | –3.4 |
| 9 | –4.2 |
| 11 | –32.2 |
